# The Influence of Maternal and Household Resources, and Parental Psychosocial Child Stimulation on Early Childhood Development: A Cross-Sectional Study of Children 36–59 Months in Honduras

**DOI:** 10.3390/ijerph15050926

**Published:** 2018-05-07

**Authors:** Helga Bjørnøy Urke, Mariela Contreras, Dennis Juma Matanda

**Affiliations:** 1 Department of Health Promotion and Development, University of Bergen, 5020 Bergen, Norway; 2 GOAL Honduras, 11101 Tegucigalpa, Honduras; mcontreras@hn.goal.ie; 3 Department of Women’s and Children’s Health, International Maternal and Child Health (IMCH), Uppsala University, 751 85 Uppsala, Sweden; 4 Population Council, Avenue 5, Rose Avenue, P.O. Box 17643-00500, Nairobi, Kenya; dmatanda@popcouncil.org

**Keywords:** early childhood development, nurturing care, DHS, Honduras

## Abstract

Optimal early childhood development (ECD) is currently jeopardized for more than 250 million children under five in low- and middle-income countries. The Sustainable Development Goals has called for a renewed emphasis on children’s wellbeing, encompassing a holistic approach that ensures nurturing care to facilitate optimal child development. In vulnerable contexts, the extent of a family’s available resources can influence a child’s potential of reaching its optimal development. Few studies have examined these relationships in low- and middle-income countries using nationally representative samples. The present paper explored the relationships between maternal and paternal psychosocial stimulation of the child as well as maternal and household resources and ECD among 2729 children 36–59 months old in Honduras. Data from the Demographic and Health Surveys conducted in 2011–2012 was used. Adjusted logistic regression analyses showed that maternal psychosocial stimulation was positively and significantly associated with ECD in the full, rural, and lowest wealth quintile samples. These findings underscore the importance of maternal engagement in facilitating ECD but also highlight the role of context when designing tailored interventions to improve ECD.

## 1. Introduction

Optimal early childhood development (ECD) is currently jeopardized for more than 250 million children under five in low- and middle-income countries [1,2]. Suboptimal development is associated with a range of unfavorable outcomes such as childhood stunting [3], lower adult educational attainment [4], and lower wages in adulthood [5]. An emerging interest in and emphasis on the importance of ensuring ECD has been noted in research, practice, and politics [1]. In particular, attention to ECD has been recognized as the foundation underpinning the achievement of several Sustainable Development Goals [6]. Black et al. [1] have proposed a theoretical life course conceptual framework of ECD in which ECD is contingent on factors at different levels: nurturing care; an enabling environment for the caregiver, family and community; and social, economic, political, climatic, and cultural contexts.

Nurturing care comprises a wide range of essential care dimensions related to health, nutrition, security and safety, responsive caregiving, and early learning. An enabling environment refers to parental, family, household, and community characteristics such as parental education, parental health, prenatal, and postnatal care. The social, economic, political and cultural contexts refer to broader systems. For example, cultural and geographical characteristics vary between and within countries, e.g., between urban and rural areas. This model implies that, in contexts with poorer systems, with fewer enabling environments and resources, as well as limited nurturing care, the chances of children achieving their developmental potential are severely impeded.

In the context of a focus shift from children *surviving* to children *thriving*, nurturing care for ECD is currently attracting increasing interest [1]. Whereas aspects of nurturing care related to health and nutrition have received substantial coverage in low- and middle-income contexts [7], less attention has been given to psychosocial aspects of nurturing care in these contexts such as responsive caregiving and early learning. Paxson and Schady [8] found a measure of parenting quality (i.e., responsivity, punitiveness, and time spent reading to the child) to be associated with cognitive development in children under five in economically poor households in Ecuador. Other studies in LMICs have documented the effectiveness of psychosocial stimulation on improved child development [9,10,11,12,13,14,15]. The literature has strongly focused on the interaction between mother and child, with less attention devoted to the role of the father. A recent study using Multiple Indicator Cluster Surveys (MICS) data from 38 LMICs assessed the associations between paternal psychosocial stimulation as measured by the Family Care Indicator (FCI) [16] and ECD [15]; results suggested that paternal psychosocial stimulation was positively associated with higher ECDI scores. The present paper focuses on the role of both maternal and paternal psychosocial stimulation, which includes cognitive and socioemotional stimulation, key factors for optimal childhood development [14].

Positive associations between an enabling environment, such as socioeconomic status (SES) and various aspects of ECD, have been documented [5,17]. In their literature review of SES and child development, Bradley and Corwyn [17] concluded that better SES, in the form of higher income and higher parental (particularly maternal) education, was associated with a range of child development outcomes, including improved cognitive achievement and socioemotional development. Although much of this literature is based on samples from western contexts, predominantly from the U.S. [17], similar patterns have also been identified elsewhere, including the Latin American region [5,8]. In their study of Ecuadorian preschool children, Paxson and Schady [8] also found that higher household wealth and higher levels of parental education were associated with higher scores on a measure of early cognitive development.

Existing studies conducted on determinants of ECD in LMICs have been characterized by small and selected samples [8,18]. Further, the scarcity of studies on ECD may be partly attributable to the lack of easily implemented global indicators for ECD. To measure ECD, some studies have used validated but significantly resource-intensive measures, such as the Bayley Scales of Infant Development [19], the Woodcock-Johnson tests, the MacArthur Communicative Development Inventories, among others [20]. Such measures are difficult to apply to large nationally representative surveys. Over the past decade, the development for such a gauge has resulted in the Early Childhood Development Indicator (ECDI). Similarly, assessment of responsive caregiving with appropriate indicators has been challenging; however, work to develop a valid indicator for use in surveys has resulted in the FCI, largely based on the HOME Inventory [16]. With increasing availability of measures in large international databases like the MICS and the Demographic and Health Surveys (DHS), research in LMICs on associations in the life course model [1] has been facilitated to a larger extent. The development of measures of psychosocial stimulation and ECD for use in international population-based surveys both calls for and enables research on these associations on a broader scale. Such research will contribute to a firmer knowledge base regarding aspects of ECD, which, in turn, can inform program development and policy. The present paper aims to contribute to this knowledge base using nationally representative DHS data from Honduras.

Honduras is a lower-middle-income country in Central America [21]. Few studies have been published on Honduras’ maternal and household resources, parental psychosocial stimulation and ECD using population-based data. Nevertheless, the government is currently implementing a nationwide ECD strategy, which calls for additional evidence-based knowledge on correlates of ECD.

This study assesses the relationship between psychosocial stimulation provided by the mother and father and ECD as well as the interrelationship among maternal and household resources (education, literacy, decision-making power, and household wealth) and ECD in Honduras. We hypothesize that children who receive more psychosocial stimulation score higher on ECD than children who receive less of such care. We further hypothesize that children of mothers with fewer resources available score lower on ECD than children of mothers with increased access to such resources.

## 2. Materials and Methods

### 2.1. Data and Sample

The study was based on the nationally representative data derived from the 2011–12 round of DHS in Honduras [22]. DHS data were collected through a stratified two-stage cluster sampling design. Data collection clusters were selected from the national population census frame; households were listed and randomly sampled according to these clusters. Households were then visited by field workers who invited household heads, including eligible women and men, to participate in the survey [22]. The sample of the present study was comprised of 2786 children 36–59 months old with mothers aged 15–49 years. All children with data on the ECD measure were included in the study, but children not living with the mother were excluded (*n* = 8), as well as multiple birth children (*n* = 42).The final weighted sample was 2736.

### 2.2. Measures

#### 2.2.1. ECD

ECD was assessed using the ECDI developed by UNICEF [23] and based on recommendations by Zill and Ziv [24]. This measure is a 10-item scale consisting of four domains: (1) language/cognitive, (2) physical, (3) socio-emotional, and (4) approaches to learning. These domains are then combined into a dichotomous early child development measure (See Supplemental Table S1 for details). Internal consistency for the combination of the ten items before further dichotomization in this study was fair, with a Cronbach’s alpha of α = 0.41. A recent study using the ECDI aggregated for 38 LMIC observed a fair Cronbach’s alpha value of α = 0.55 [15]. The number of items in each domain of the ECDI varies; each child is categorized as *on track* or *not on track* in each of the domains. The four domains are defined accordingly. (1) Language/cognitive: identify letters, read simple words, identify numbers (*on track* if 2/3 items achieve a yes response). Physical: can pick up item with two fingers and does not often feel sick (*on track* if 1/2 items achieve a yes response). Socioemotional: gets along with other children, does not kick or bite other children (*on track* if 1/2 items achieve a yes response). Approaches to learning: can follow simple instructions, can perform simple tasks independently, is not easily distracted (*on track* if 2/3 items achieve a yes response). Each domain was summarized into an early childhood development variable and, as recommended by the developers of the index [23], dichotomized into *not on track* and *on track. On track* refers to *on track in 3/4 of the domains.* The 10-item ECDI has been validated in several countries [23].

#### 2.2.2. Psychosocial Stimulation

Psychosocial stimulation was measured with two variables which indicated paternal and maternal activity engagement with the child during the seven days prior to the interview. These variables were the sum of scores which entailed six items that asked whether the father and/or the mother engaged with the child in specific activities (reading, storytelling, playing, drawing, playing, and going outside), with response categories of *no* and *yes*. The measures were adapted versions of the activity engagement dimension of the FCI, which is a population-based measure developed from the HOME Inventory instrument [16]. Cronbach’s alpha values for the variables in this study were α = 0.73 and α = 0.83 for maternal and paternal psychosocial stimulation, respectively.

#### 2.2.3. Maternal and Household Resources

Maternal education included the following categories: *no or incomplete primary education, complete primary education,* and *secondary or higher education.* Household wealth was measured with the DHS wealth index quintiles, a composite measure including housing material, assets, and facilities. The first quintile reflected the lowest levels of wealth, while the fifth quintile reflected the highest level of wealth [25]. Maternal literacy was assessed on the basis of the success (*literate*) or failure (*illiterate*) of the respondent to read parts or the entirety of a specific sentence presented to her. Maternal decision-making power was assessed based on a scale consisting of four dichotomous items addressing *no* or *partial/full involvement* in decision making on household and personal issues (own healthcare, large household purchases, visits to family and friends, and how to spend own earnings). In addition, whether the child attended an early learn0ing program (*no* or *yes*) was included in the assessment.

#### 2.2.4. Covariates

Covariates included maternal age in years, maternal marital status (*never lived with a partner, married/living with a partner,* and *divorced/widowed/no longer living with partner*), child age in months, child sex, number of children under the age of five living in the household, geographic region (*Northern (Atlántida, Cortés, Islas de la Bahía, and Yoro), Southern (Choluteca, El Paraíso, and Valle), Western (Copán, Intibucá, La Paz, Lempira, Ocotepeque, and Santa Bárbara), Central (Comayagua and Francisco Morazán)* and *Eastern (Colón, Gracias a Dios, and Olancho)*, and place of residence categorized as *urban* or *rural* based on assessments made by DHS [22].

### 2.3. Statistical Analysis

Regression analyses were performed using logistic regression in which ECD was regressed on maternal psychosocial stimulation and on each of the maternal and household resource variables. To explore the influence of context and socioeconomic factors on ECD, separate regression models were run for urban and rural samples as well as for a joint first and second as well as fourth and fifth wealth quintiles, to examine possible differences in the relationship between maternal psychosocial stimulation and ECD in these groups. Both unadjusted and adjusted analyses were run. Our final adjusted regression models included variables mentioned under the Covariates Section 2.2.4. Descriptive and regression analyses were performed in IBM SPSS Statistics 24 using the SPSS Complex module, taking account of sampling strategy.

### 2.4. Ethics

Data were collected with informed consent, and respondent anonymity was guaranteed [22]. The study questionnaires and protocols were reviewed and approved by the ICF Institutional Review Board as complying with the United States Department of Health and Human Services requirements for the “Protection of Human Subjects” (45 Code of Federal Regulations part 46) [26].

## 3. Results

Descriptive results are presented in Table 1. Mean maternal age was 29 years and mean child age was 47 months. The sample had a slight overweight of boys (53% boys compared to 47% girls). 48% of the sample lived in urban areas, and 52% lived in rural areas. In terms of ECD, the outcome of interest, 13% of children were not on track as assessed by the ECDI. The mean score for the ECDI in our sample was 5.94 (1.44). This level was similar to a recent study assessing ECD across 38 low- and middle-income countries using the same measure, which observed a total mean and standard deviation (SD) score of *M* = 5.76 (SD = 1.89) [15]. The majority of women sampled (36%) either had no education or incomplete primary education.

Results from regression analyses are presented in Table 2. Psychosocial child stimulation was statistically significant and positively associated with a child being on track with ECD in both the full and rural samples but not in the urban sample. In analyses restricted to wealth quintiles which analyzed the combined first and second quintiles as well as the combined third and fourth, psychosocial child stimulation was significantly associated with being on track with ECD in low wealth quintiles (Odds Ratio (OR) 1.14, 95% Confidence Interval (CI) 1.03, 1.25) but not significantly in the high wealth quintiles (OR 1.17, 95% CI 0.95, 1.44) (results not presented).

## 4. Discussion

This study explored the associations among psychosocial child stimulation, maternal and household resources (namely maternal education, maternal decision-making power, maternal literacy, and household wealth), and ECD among children 36–59 months old in a nationally representative sample from Honduras. These associations were explored in the full, urban, rural, poorest, and richest wealth quintiles in the data.

Maternal psychosocial child stimulation was positively and significantly associated with ECD in the full, rural, and poorest wealth quintile samples but not in the urban and higher wealth quintile samples. Specifically, in the full, rural and poorest wealth quintile samples, children who received more psychosocial stimulation were more likely to be on track with ECD, as measured by the ECDI, in comparison with children who received less of such care. Paternal psychosocial child stimulation was not significantly associated with ECD in either of the samples. The finding of a significant association of maternal psychosocial child stimulation is in accordance with our initial hypothesis regarding the direction of the relationship between psychosocial stimulation and ECD. With regard to our hypothesis about the relationship among maternal and household resources and ECD, our data did not provide the same support. With the exception of maternal decision-making power in the full sample, none of the maternal and household resources were significantly associated with ECD in regression analyses.

The findings of a positive association between maternal psychosocial stimulation and ECD in our study are in line with previous similar studies [7,8]. Further, intervention studies have found child stimulation interventions to have significant benefits to overall child development [12,13,14,27]. A community-based cluster-randomized control trial in Pakistan demonstrated that children who received responsive stimulation had substantially higher development scores on the cognitive, language, and motor scales at 12 and 24 months of age as well as on the socioemotional scale at 12 months of age compared to children in the control group [14]. A follow-up study showed that children who received responsive stimulation had significantly higher cognition, language, and motor skills at four years of age than children who did not receive responsive stimulation [12,13]. Similar results have also been reported in other randomized control trials in Uganda [12] and Jamaica [28,29].

With regard to the difference in association between maternal psychosocial child stimulation and ECD among urban and rural samples as well as wealth quintile groups, a potential explanation could be that, in urban areas and among higher wealth quintile households, other resources to facilitate ECD are more readily available. For example, the conceptual framework by Black et al. [1] underscores several aspects of nurturing care that are important for ECD, such as access to health care, a diverse diet, early learning programs, etc. With the exception of attendance in early learning programs, which did not produce a significant effect on ECD, we did not examine these factors. It is possible that the inclusion of child diet or health promotion measures could have explained more of the variance in ECD.

As already mentioned, previous similar studies have often concentrated on samples from vulnerable populations, e.g., from lower socioeconomic groups. The group differences observed in our study highlight the importance of sensitivity analyses, as they facilitate a broader understanding of variation in ECD. Such knowledge can help tailor efforts to specific groups of children to facilitate each child’s ability to reach its developmental potential.

The lack of an association among maternal and household resources and ECD in the present study was somewhat puzzling, as it contradicted previous similar research [5,8]. Potential reasons for our findings could be related to the measures included in the study. For example, the household wealth index did not directly reflect income or expenditure, which could be more relevant in the facilitation of ECD. Further, the maternal education measure may have been insufficiently sensitive to capture any variations in ECD which were dependent on education level. Also, even if education level was considered a gateway to specific knowledge or practices [30,31], e.g., regarding children’s needs which could influence ECD positively, it did not necessarily have such an indirect effect on ECD. Having data on more specific knowledge may yield different results in relation to ECD.

A shift in focus from a child merely surviving to thriving implicates research, practices, and policies that facilitate a range of nurturing care aspects. The findings in the present study support the role of parental psychosocial stimulation of children as one such method of care practice. This also has practical implications in the development of ECD efforts which facilitate such stimulation. Further, given that stunting and impaired cognitive development present similar risk factors, scaling up ECD and nutrition interventions synergistically could offer a potential way forward. The benefits of the implementation of good ECD programs in conjunction with nutritional interventions was recommended in the Maternal and Child Nutrition Lancet series in 2013 [32].

The study had several limitations that should be noted. The data were cross-sectional, limiting the possibility of making causal inferences between the variables under study. Longitudinal designs are required to more firmly establish the direction of relationships of interest in the present study.

An important strength of the present study included the examination of the relationship of both maternal and paternal psychosocial stimulation to the newly developed ECD measure in a LMIC. The literature is limited on this aspect in Latin America; accordingly, this study contributes to the literature on these associations in LMIC generally and in Latin America and Honduras in particular. Furthermore, the study was based on a nationally representative sample, also contributing to research which has often been characterized by small and selective samples.

## 5. Conclusions

ECD is key to thriving children worldwide. A broad understanding of risks and resources for optimal ECD is necessary but scarce, especially in LMICs. The present study contributes to this literature by shedding light on the role of psychosocial stimulation provided by the mother and father in different subgroups with varying levels of vulnerability. To meet the development needs of children, the role of maternal interaction with children should be emphasized. The findings call for ECD efforts that are sensitive to context and SES to ensure the most effective use of resources.

## Figures and Tables

**Table 1:** Descriptive characteristics of sample *, *n* = 2736. Honduras Demographic and Health Surveys 2011–2012.

	Variable	Mean (SD) or *n* (%)
Maternal characteristics		
	Age (years)	29.54 (6.69) **
	Relationship status	
	Never in union	101 (3.7%)
	Married or living with a partner	2223 (81.2%)
	Widowed, divorced, separated	412 (14.0%)
	Education	
	No or incomplete primary education	946 (34.6%)
	Complete primary education	861 (31.5%)
	Secondary or higher education	929 (34.0%)
	Literacy	
	Illiterate	287 (10.5%)
	Literate	2443 (89.3%)
	Missing	6 (0.2%)
	Decision-making power	
	No	86 (3.2%)
	1 issue	224 (8.2%)
	2 issues	503 (18.4%)
	3 issues	1409 (51.5%)
	Missing	513 (18.8%)
Child characteristics		
	Age (months)	47.42 (6.80) **
	Child sex	
	Male	1439 (52.6%)
	Female	1297 (47.4%)
	Early child development	
	Not on track	352 (12.9%)
	On track	2377 (86.9%)
	Missing	7 (0.3%)
	Child attends early learning program	
	No	2215 (80.9%)
	Yes	516 (18.9%)
	Missing	5 (0.2%)
Household characteristics		
	Number of children <5 years living in household	1.59 (0.81) **
	Household wealth	
	First quintile	618 (22.6%)
	Second quintile	549 (20.1%)
	Third quintile	536 (19.6%)
	Fourth quintile	592 (21.6%)
	Fifth quintile	441 (16.1%)
	Geographic region	
	Northern	896 (32.7%)
	Southern	320 (11.7%)
	Western	596 (21.8%)
	Central	621 (22.7%)
	Eastern	303 (11.1%)
	Place of residence	
	Urban	1305 (47.7%)
	Rural	1431 (52.3%)
	Maternal participation in activity with child	3.32 (1.86) **
	Paternal participation in activity with child	1.81 (1.96) **

* Sample is weighted. ** Mean (SD).

**Table 2:** Fully adjusted ^1^ models examining maternal and household resources, maternal and paternal psychosocial stimulation of child, and early childhood development, *n* = 2736. Honduras Demographic and Health Surveys 2011–2012.

	Full Sample (*n* = 2736)		Urban Sample (*n* = 1305)		Rural Sample (*n* = 1431)	
Variables	B (S.E.)	OR (95% CI)	B (S.E.)	OR (95% CI)	B (S.E.)	OR (95% CI)
Maternal engagement in activity with child	0.13 (0.04)	1.14 (1.04, 1.24) ***	0.06 (0.08)	1.07 (0.90, 1.25)	0.17 (0.06)	1.18 (1.06, 1.30) ***
Paternal engagement in activity with child	0.00 (0.04)	1.00 (0.92, 1.09)	0.02 (0.08)	1.02 (0.88, 1.19)	−0.01 (0.05)	0.99 (0.90, 1.09)
Child attends early learning program (ref = No)						
Yes	0.07 (0.20)	1.07 (0.72, 1.58)	−0.02 (0.37)	0.98 (0.48, 2.02)	0.23 (0.24)	1.25 (0.79, 2.00)
Maternal education (ref = No or incomplete primary education)						
Complete primary education	0.20 (0.18)	1.02 (0.71,1.46)	0.03 (0.44)	1.03 (0.44, 2.44)	0.44 (0.29)	1.02 (0.68, 1.52)
Secondary or higher education	−0.23 (0.23)	0.80 (0.50, 1.26)	−0.02 (0.42)	0.98 (0.43, 2.22)	0.46 (0.28)	0.65 (0.37, 1.13)
Literacy (ref = Illiterate)						
Literate	0.27 (0.22)	1.30 (0.84, 2.02)	−0.43 (0.74)	0.65 (0.15, 2.81)	0.37 (0.24)	1.45 (0.91, 2.30)
Decision-making power (ref = 0 issues)						
1 issue	−0.46 (0.37)	0.63 (0.30, 1.35)	−0.56 (1.19)	0.57 (0.06, 5.94)	−0.42 (0.42)	0.66 (0.29, 1.49)
2 issues	−0.73 (0.39)	0.48 (0.24, 0.97) *	−1.07 (1.17)	0.35 (0.04, 3.41)	−0.63 (0.37)	0.53 (0.26, 1.10)
3 issues	−0.51 (0.35)	0.60 (0.30, 1.18)	−0.39 (1.14)	0.68 (0.07, 6.36)	−0.61 (0.36)	0.54 (0.27, 1.10)
Household wealth (ref = First quintile)						
Second quintile	−0.14 (0.17)	0.87 (0.63, 1.20)	−0.57 (0.66)	0.55 (0.15, 2.01)	−0.09 (0.18)	0.91 (0.64, 1.30)
Third quintile	−0.30 (0.24)	1.34 (0.84, 2.14)	0.22 (0.78)	1.25 (0.27, 5,74)	0.33 (0.26)	1.39 (0.83, 2.31)
Fourth quintile	−0.50 (0.30)	1.64 (0.91, 2.95)	0.65 (0.76)	1.91 (0.43, 8.52)	0.40 (0.35)	1.49 (0.75, 2.95)
Fifth quintile	−0.75 (0.41)	2.11 (0.94, 4.75)	1.00 (0.83)	2.72 (0.53, 13.98)	0.15 (0.57)	1.16 (0.38, 3.559)

^1^ All analyses are adjusted for maternal age, maternal marital status, geographic region, number of children <5 years living in household, and child age and sex. r2 model fit estimates = 0.03–0.06, 0.03–0.07, and 0.03–0.05 for full, urban and rural sample respectively. B indicates regression coefficient; S.E. indicates standard error of B; OR indicates odds ratio; CI indicates confidence interval; ref indicates reference category. OR with CI not including 1 are indicated in bold. *** *p* ≤ 0.001, * *p* ≤ 0.05.

## Supplementary Materials

The following are available online at http://www.mdpi.com/1660-4601/15/5/926/s1, Table S1: Early Childhood Development Index.
