# Supplementary material for: The Influence of Maternal and Household Resources, and Parental Psychosocial Child Stimulation on Early Childhood Development: A Cross-Sectional Study of Children 36–59 Months in Honduras
Source: Int J Environ Res Public Health. 2018 May 7;15(5):926. doi: 10.3390/ijerph15050926 (PMC5981965; doi:10.3390/ijerph15050926)
Supplement: Supplementary file 1 [file ijerph-15-00926-s001.pdf]

The Influence of Maternal and Household Resources, and Parental Psychosocial Child Stimulation on Early Childhood Development: A Cross-Sectional Study of Children 36–59 Months in Honduras

Helga Bjørnøy Urke, Mariela Contreras and Dennis Juma Matanda

Supplemental Material

Table S1. Early Childhood Development Index *.

Domain	Questions	Coding (no/yes)
Language/Cognitive	Can your child identify or name at least 10 letters of the alphabet?	
	Can your child read at least 4 simple, popular words?	
	Does your child know the name and recognize the symbol of all numbers from 1 to 10?	
Physical	Can your child pick up a small object with two fingers, like a stick or a rock from the ground?	
	Is your child sometimes too sick to play?	Reverse coded
Socio-emotional	Does your child get along well with other children?	
	Does your child kick, bite, or hit other children or adults?	Reverse coded
	Does your child get easily distracted?	Reverse coded
Approaches to learning	Does your child follow simple directions on how to do something?	
	When given something to do, is your child able to do it independently?	

* Loizillon, A.; Petrowski, N.; Britto, P.; Cappa, C. *Development of the Early Childhood Development Index in MICS Surveys*; UNICEF: New York, NY, USA, 2017.
